# Changing epidemiology of shigellosis in Taiwan, 2010-2019: an emerging threat to HIV-infected patients and men who have sex with men

**DOI:** 10.1080/22221751.2022.2031309

**Published:** 2022-02-10

**Authors:** Chin-Shiang Tsai, Kuan-Yin Lin, Bo-Huang Liou, Chien-Shun Chiou, Yi-Chun Lin, Yuan-Ti Lee, Chia-Jui Yang, Hung-Jen Tang, Ying-Shu Liao, Chun-Eng Liu, Chen-Hsiang Lee, Po-Liang Lu, Sung-Hsi Huang, Chien-Ching Hung, Wen-Chien Ko, on behalf of the Taiwan HIV Study Group

**Affiliations:** a Institute of Clinical Medicine, College of Medicine, National Cheng Kung University, Tainan, Taiwan; b Department of Medicine, National Cheng Kung University Hospital, College of Medicine, National Cheng Kung University, Tainan, Taiwan; c Department of Internal Medicine, National Cheng Kung University Hospital, Dou-Liou Branch, College of Medicine, National Cheng Kung University, Yunlin, Taiwan; d Department of Internal Medicine, National Taiwan University Hospital and National Taiwan University Hospital, Taipei, Taiwan; e Department of Internal Medicine, Hsinchu Mackay Memorial Hospital, Hsinchu City, Taiwan; f Centre for Diagnostics and Vaccine Development, Centres for Disease Control, Taichung, Taiwan; g Department of Infectious Diseases, Taoyuan General Hospital, Ministry of Health and Welfare, Taoyuan, Taiwan; h Department of Internal Medicine, Chung Shan Medical University Hospital, Taichung, Taiwan; i School of Medicine, Chung Shan Medical University, Taichung, Taiwan; j School of Medicine, National Yang-Ming University, Taipei, Taiwan; k Department of Internal Medicine, Far Eastern Memorial Hospital, New Taipei City, Taiwan; l Department of Internal Medicine, Chi Mei Medical Centre, Tainan, Taiwan; m Department of Health and Nutrition, Chia Nan University of Pharmacy and Sciences, Tainan, Taiwan; n Department of Internal Medicine, Changhua Christian Hospital, Changhua, Taiwan; o Department of Internal Medicine, Kaohsiung Chang Gung Memorial Hospital and Chang Gung University College of Medicine, Kaohsiung, Taiwan; p Department of Internal Medicine, Kaohsiung Medical University Hospital and College of Medicine, Kaohsiung Medical University, Kaohsiung, Taiwan; q Department of Internal Medicine, National Taiwan University Hospital Hsin-Chu Branch, Hsin-Chu, Taiwan; r Department of Tropical Medicine and Parasitology, National Taiwan University College of Medicine, Taipei, Taiwan; s Department of Medical Research, China Medical University Hospital, Taichung, Taiwan; t China Medical University, Taichung, Taiwan; u Department of Medicine, College of Medicine, National Cheng Kung University, Tainan, Taiwan

**Keywords:** Bacillary dysentery, sexually transmitted disease, oro-anal sex, antimicrobial resistance, fluoroquinolones

## Abstract

Shigellosis appears to increase in certain at-risk populations in developed countries. Based on the nationwide surveillance, the annual incidence of shigellosis in Taiwan (1999-2019) was 0.38-5.77 cases per 100,000 people. Indigenous shigellosis has mostly affected men who have sex with men (MSM) and people living with HIV (PLWH) since 2015. In this retrospective study, compared with those diagnosed before 2015, indigenous cases diagnosed during 2015–2019 mostly occurred in male adults (96.0% vs 47.1%, *P *< 0.001), with a longer hospital stay (median 5.0 vs 3.5 days, *P *= 0.029) and different coinfections. The predominant strains in 2015 and 2016 were ciprofloxacin-resistant *Shigella sonnei* and azithromycin non-susceptible *Shigella flexneri* (*S. flexneri*) 3a, which had been replaced by ciprofloxacin-resistant *S. flexneri* 2a since 2018. Notably, six indigenous cases were caused by cefotaxime-resistant *S. flexneri*. Inappropriate use of empiric antibiotic treatment was common. In conclusion, there is an ongoing spread of ciprofloxacin-resistant shigellosis among PLWH and MSM and cefotaxime-resistant *S. flexneri* is an emerging threat in Taiwan.

## Introduction

*Shigella* is a non-spore-forming, non-motile, Gram-negative bacterium, and is well-known as an etiologic agent of bacillary dysentery. The genus includes four serogroups with multiple serotypes: *S. dysenteriae*, (serogroup A), *S. flexneri* (serogroup B), *S. boydii*, (serogroup C), and *S. sonnei* (serogroup D) [1]. *Shigella* spp. can be transmitted by faecal-oral route with high infectivity and remains one of the important enteropathogens in the low- and middle-income countries (LMIC), especially in paediatric patients [2].

Shigellosis is a public health problem in LMIC and has occasionally been reported among men who have sex with men (MSM) or people living with HIV (PLWH) as a sexually transmitted disease [3–6]. Recently shigellosis has caused several outbreaks among MSM [7–11], especially those using recreational drugs for sex (“chemsex”) [12–14], in which *S. flexneri* and *S. sonnei* have been the predominant species [15]. Moreover, a global threat of decreased antimicrobial susceptibility of *Shigella* isolates has emerged and caused concerns about the optimal treatment of shigellosis [16–20]. Recently, there were reports of domestic transmission of fluoroquinolone-resistant *S. sonnei* and azithromycin non-susceptible *S. flexneri* 3a among MSM in Taiwan [21,22]. However, information was limited on the incidence of shigellosis among the entire population, the trends of affected populations by the national surveillance system, and clinical characteristics and the outcome of shigellosis among MSM and PLWH in Taiwan. Therefore, we performed a multicentre retrospective study to delineate the evolving epidemiology of shigellosis and antimicrobial resistance profile of *Shigella* species between 2010 and 2019, especially among MSM and PLWH.

## Methods

### Study setting and population

In Taiwan, shigellosis is nationally notifiable through the web-based Taiwan Centres for Disease Control (TCDC)-operated Notifiable Disease Surveillance System (NDSS), and public health investigations are conducted, including environmental and water supply investigations [23,24], and all *Shigella* isolates are required to be submitted to TCDC for confirmation.

In this study, the reported shigellosis cases on the NDSS in Taiwan during 1999–2019 were reviewed. A retrospective study was conducted to include individuals who received a diagnosis of shigellosis and sought care at 11 major hospitals around Taiwan from January 2010 to December 2019 (Supplementary Figure 1). Clinical information of individuals with shigellosis was retrieved from the electronic medical records and recorded in a standardized case record form, which included age, sex at birth, sexual orientation, dates of admission or hospital visit, presenting symptoms (such as duration of diarrhoea before seeking medical attention, fever, abdominal pain, bloody diarrhoea, tenesmus, vomiting, or altered mental status), exposure to uncooked food, travel history to endemic areas, substance abuse, HIV serostatus, antibiotic therapy, and clinical outcome (such as recurrence and the length of hospital stay). Concurrent sexually transmitted diseases (STDs), such as gonorrhoea, chlamydia, or syphilis, or hepatitis coinfections were also recorded when shigellosis was diagnosed. For PLWH, their recent CD4 count and plasma HIV RNA load were recorded.

### Definitions

The indigenous cases of shigellosis were defined as shigellosis occurring in individuals without recent travel to endemic areas; and the imported cases were defined as shigellosis occurring in individuals with recent travel to endemic areas. Chronic diarrhoea was defined as diarrhoea for ≥14 days. Hepatitis A infection was defined by the presence of serum hepatitis A virus immunoglobulin G (HAV IgG) without previous vaccination. Chronic hepatitis B virus (HBV) infection was defined as being seropositive for hepatitis B virus surface antigen (HBsAg) for six months or longer, and hepatitis C infection as being seropositive for hepatitis C virus. Amoebiasis was defined by being tested positive for *Entamoeba histolytica*, seropositivity for indirect haemagglutinin assay (IHA), or histopathology, with or without consistent symptoms. Diagnosis of gonorrhoea was made by microbiologic cultures or nucleic-acid amplification tests of relevant clinical specimens, while chlamydia infection was diagnosed by nucleic-acid amplification tests. Syphilis was diagnosed based on consistent clinical symptoms and signs plus an elevated rapid plasma reagin (RPR) titre with a reactive *Treponema pallidum* particle agglutination (TPPA) assay.

The definition for appropriate antibiotic use for shigellosis was defined as the fulfilment of all the following criteria: (i) the route and dosage of antimicrobial agents were administered as recommended in the Sanford Guide; (ii) the causative isolate was susceptible to the administrated antimicrobial agent *in vitro* based on the contemporary breakpoints of the Clinical and Laboratory Standards Institute (CLSI) (*23*); and (iii) the time from hospital arrival or symptom onset upon admission to the prescription of an appropriate antibiotic should not be longer than 48 h.

### Laboratory investigations

The results of any laboratory data during the period between two days before and after the diagnosis of shigellosis were recorded, including white blood cell (WBC) counts and differential, serum creatinine, and serum alanine aminotransferase. The results of microscopic examinations of faecal samples (such as WBCs, red blood cells, ova, or parasites) were also collected.

Antimicrobial susceptibility tests were performed using a disk diffusion method by following the CLSI guidelines [25], and the drugs tested were at the discretion of each participating hospital. The *Shigella* colonies were used for slide agglutination tests with commercially available polyclonal antisera (Denka Seiken Company, Japan) that cover the common species or serotypes. To perform serotyping, about 20 μl of antigen suspension and 20 μl of antiserum were added on a slide and mixed with the microbiological loop. The slide was tilted back and forth for about 1 min and was put in a dark background to observe for agglutination.

### Statistical analysis

All statistical analyses were performed using the statistical software IBM SPSS Statistics for Windows, version 22.0 (IBM Corp., USA). The chi-square test and Fisher’s exact analysis were used to compare categorical variables. An independent Student *t*-test was applied to compare continuous variables between groups. Mann–Whitney U test was performed to compare two independent groups when the dependent variable was either ordinal or continuous, without normal distribution. A two-tailed *P* value of less than 0.05 was considered to be statistically significant.

### Ethics statement

The study was approved by the Research Ethics Committees of National Taiwan University Hospital [registration number, 201003112R], National Taiwan University Hospital Hsinchu Branch [105-017-F] and Far Eastern Memorial Hospital [105040-F], and Institutional Review Boards of Taoyuan General Hospital [TYGH103011], Hsinchu Mackay Memorial Hospital [18MMHIS008e], Chung Shan Medical University Hospital [CS14034], Changhua Christian Hospital [160408], National Cheng Kung University Hospital [B-BR-109-032], Chi Mei Medical Centre [10505-002], Kaohsiung Medical University Hospital [KMUH-IRB-20110040], and Kaohsiung Chang Gung Memorial Hospital [202001763B0C501]. The informed consent was waived. This study was conducted according to the principles expressed in the Declaration of Helsinki.

## Results

### Nationwide surveillance of shigellosis in Taiwan, 1999–2019

**Figure 1.**
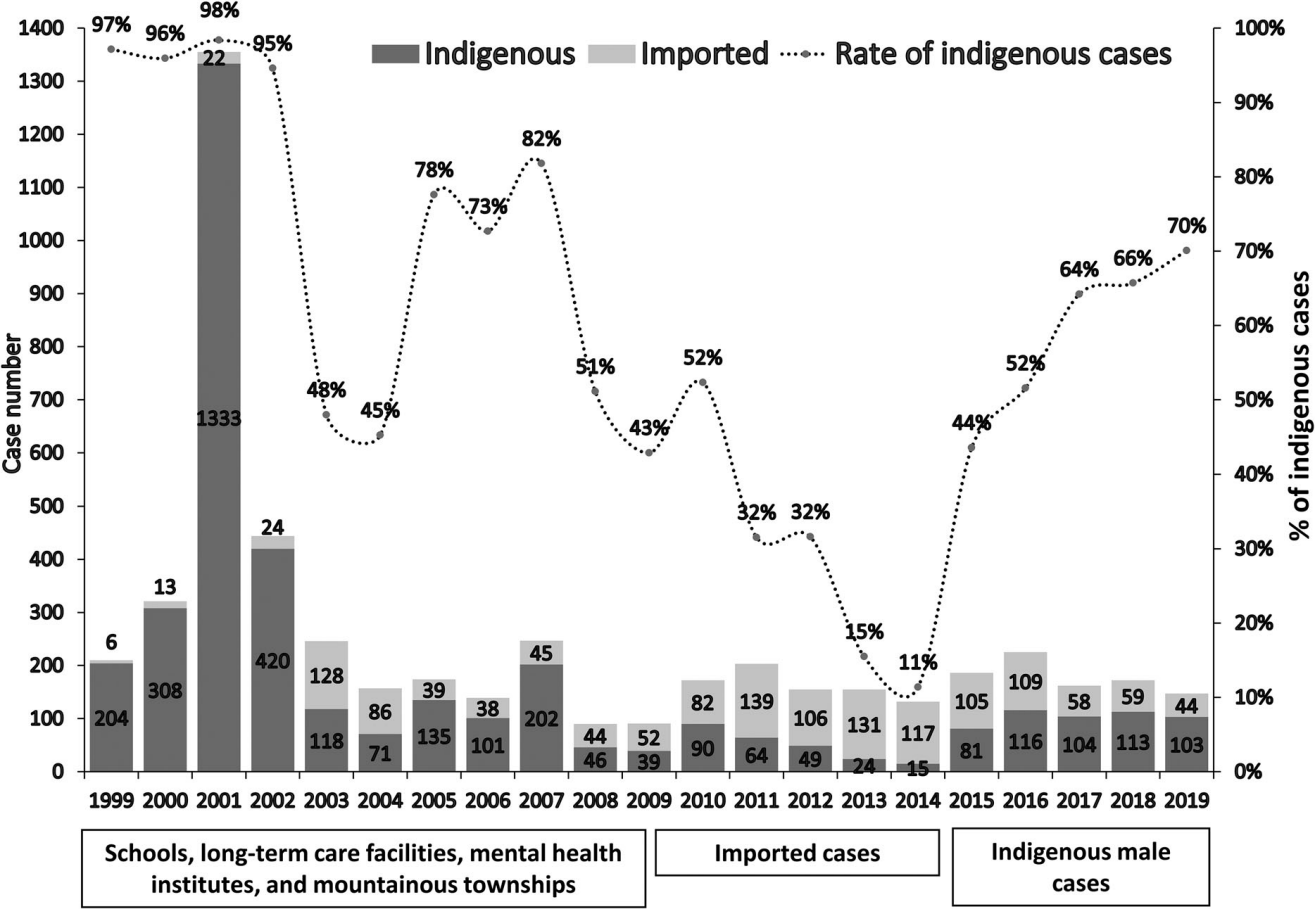
Trends of shigellosis and possible places of acquisition (indigenous or imported cases) in Taiwan from 1999 to 2019.

According to the statistics of NDSS, the annual incidence of shigellosis in Taiwan from 1999 to 2019 was approximately 0.38-5.77 cases per 100,000 people. Before 2010, indigenous cases of shigellosis accounted for most of the cases. Most of the indigenous cases occurred in mountainous townships; and several outbreaks had been reported in schools, mental health institutions, and long-term care facilities (LTCFs) in urban areas (Figure 1). During 2010-2014, there was an increasing number of imported cases, while the number of indigenous cases significantly decreased; thus, the proportion of indigenous shigellosis had declined from 52% in 2010–11% in 2014. Since 2015 a surge of indigenous cases of shigellosis has been noted, accounting for 70% of the cases of shigellosis diagnosed in 2019. Most of the indigenous cases of shigellosis diagnosed after 2015 involved male patients without a history of travel to endemic areas (Figure 1); moreover, more than 50% of the cases occurred in PLWH [26].

### Changing characteristics of shigellosis in Taiwan, 2010–2019

To investigate the characteristics of indigenous cases of shigellosis before and after 2015, we included 201 confirmed cases of shigellosis reported by 11 major hospitals around Taiwan from January 2010 to December 2019. The total case number from each hospital ranged from 2 to 53 (Supplementary Figure 1). Of the 201 cases, 189 (94.0%) were *Shigella* dysentery, 10 (5.0%) *Shigella* bacteraemia, and one both dysentery and bacteraemia; and the remaining three cases were diagnosed by either rectal swab (2 cases) or wound culture from an anal abscess (1 case). Among the cases, 83.6% (168/201) occurred in individuals without a recent history of travel abroad before the onset of symptoms and were regarded as indigenously acquired. Among the indigenous cases, males were predominant (91.1%, 153/168) and the majority (94.8%, 145/153) were diagnosed during 2015–2019 (Supplementary Figure 2). The clinical characteristics of the patients diagnosed with indigenous cases of shigellosis during 2010–2014 (Group A) and those during 2015–1019 (Group B) are summarized in Table 1. The age distribution was different between the two groups (*P *= 0.002). More than half of the affected individuals in Group A were aged <18 years, while in Group B they were mostly male aged 18–65 years. In addition, recent ingestion of uncooked food was more common in Group A (*P *= 0.009), and none in Group A had prior STDs. On the contrary, most patients in Group B were PLWH. Except for a longer hospital stay for Group B (*P *= 0.029), there were no significant differences in clinical manifestations, such as fever, abdominal pain, tenesmus, bloody diarrhoea, or chronic diarrhoea, between the two groups (Table 1).

**Table 1.** Characteristics of 168 patients with indigenous shigellosis.

Clinical variables		Group A 2010–2014 (n = 17)	Group B 2015–2019 (n = 151)	*P* value
Age, median (interquartile range), years		9.68 (5.18-35.18)	31.13 (26.65-38.88)	0.002
<18		10 (58.8)	3 (2.0)	
18–40		5 (29.4)	118 (78.1)	
41–65		1 (5.9)	27 (17.9)	
>65		1 (5.9)	3 (2.0)	
Male sex		8 (47.1)	145 (96.0)	<0.001
Men who have sex with men		0 (0)	97 (64.2)	<0.001
HIV infection		0 (0)	115 (76.2)	<0.001
Substance or recreational drug use†		0 (0)	24 (15.9)	0.078
Exposure to uncooked food		4 (23.5)	9 (6.0)	0.009
Clinical manifestations				
Fever		12 (70.6)	97 (64.2)	0.603
Abdominal pain		10 (58.8)	85 (56.3)	1.000
Diarrhoea		17 (100)	127 (84.1)	0.136
Duration of diarrhoea before visit		2.0 (0.5-3.5)	2.0 (1.0-5.0)	0.267
Diarrhoea ≥ 14 days before visit		0 (0)	18 (11.9)	0.221
Bloody diarrhoea		6 (35.3)	41 (27.2)	0.569
Tenesmus		0 (0)	5 (3.3)	1.000
Vomiting		4 (23.5)	22 (14.6)	0.305
Altered mental status		0 (0)	3 (2.0)	1.000
Hospitalization		8 (47.0)	70 (46.4)	1.000
Length of hospital stay (days)		3.5 (3.0-4.0)	5.0 (4.0-8.0)	0.029
Recurrence		0 (0)	5 (3.3)	1.000
Coinfections		2 (11.8)	15 (9.9)	0.684
**Laboratory findings**		** **	** **	** **
White blood cell count (/mm^3^)		13.03 (9.7-19.13)	8.83 (6.01-12.06)	0.001
Bandemia >10%		3 (17.6)	10 (6.6)	0.209
Acute kidney injury		1 (5.9)	20 (13.2)	0.699
Positive faecal leukocytes		11 (64.7)	89 (58.9)	0.796
Positive faecal reticulocytes		10 (58.8)	92 (60.9)	1.000
Stool ova or parasites		0 (0)	5 (3.3)	1.000

*Data are expressed as the case number (%). Categorical data are presented as counts and percentages and continuous variables as the means (± standard deviation) or median (interquartile range), if non-normally distributed.

†Substance or recreational drugs: opiates, amphetamine, ecstasy, and club drugs.

#Ova and parasite: microscopic finding of parasite ova, cysts, or trophozoites.

**Figure 2.**
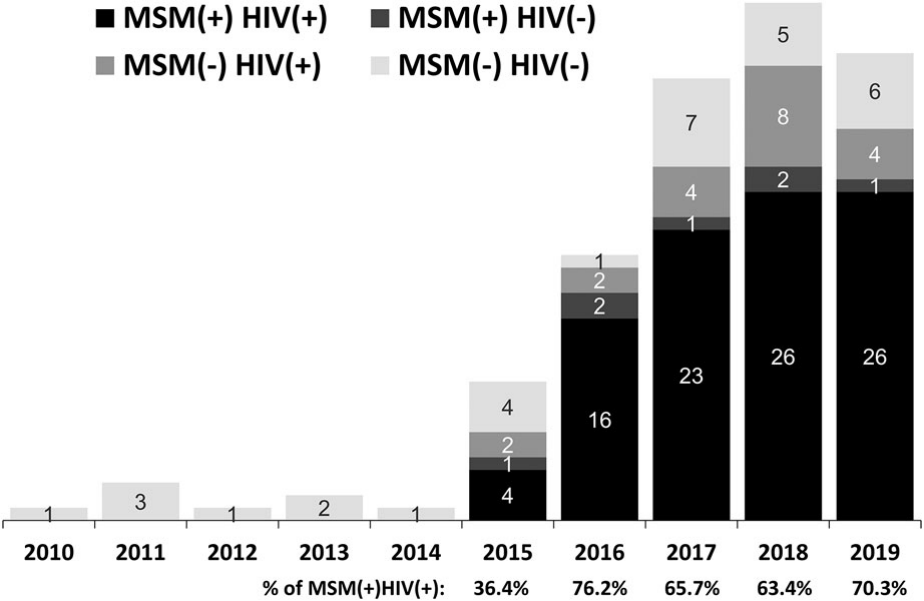
Annual case numbers of indigenous shigellosis from 2010 to 2019, categorized by HIV infection and men who have sex with men (MSM).

All 115 PLWH with indigenous cases of shigellosis were males, predominantly MSM (supplementary Table 1 and Figure 1). The annual proportion of PLWH among MSM increased from 36.4% in 2015–70.3% in 2019 (Figure 2). Of the PLWH with shigellosis, less than half had an undetectable plasma HIV RNA load (<50 copies/mL) and some had at least one coinfection, including HAV infection, chronic HBV infection, or amoebiasis (supplementary Table 1). Of note, more than 80% had had a history of syphilis. None of the patients without HIV infection had a history of STD or viral hepatitis coinfection. Concurrent infections at the onset of shigellosis varied greatly between non-HIV, non-MSM patients and PLWH or MSM. There were four (23.5%) patients with rotavirus gastroenteritis in 2010–2014 (Group A), while diverse gastrointestinal infections (including amoebic, cytomegalovirus, or *Campylobacter* colitis, and intestinal giardiasis), *Campylobacter* bacteraemia, and opportunistic infections (such as tuberculosis or candidiasis) were concurrently diagnosed in the patients in 2015–2019 (Group B). Only one patient in Group B had concurrent rotavirus gastroenteritis (Supplementary Table 2).

### Species, serotypes, and antimicrobial susceptibility of the *Shigella* isolates

**Figure 3.**
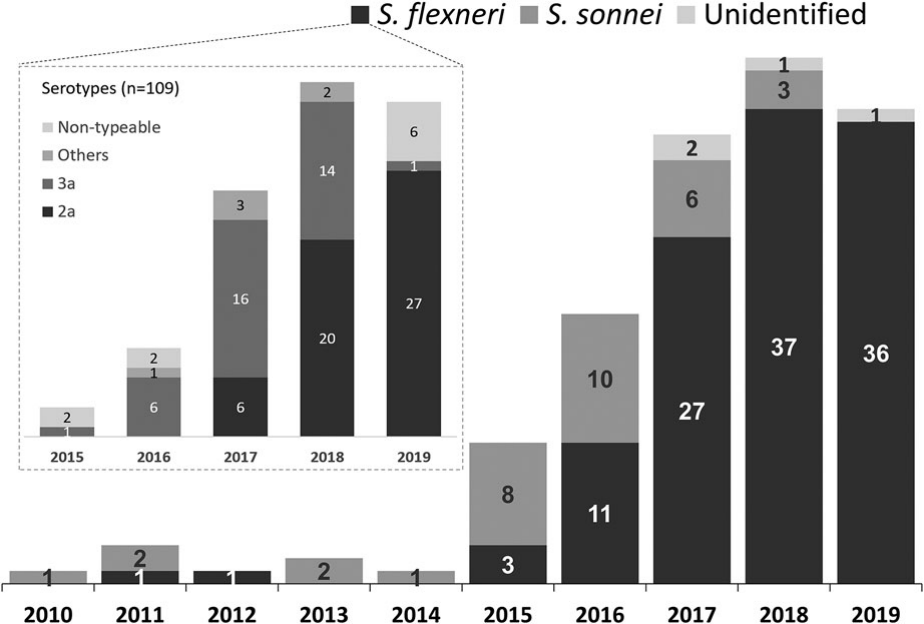
Species distribution of 153 *Shigella* isolates among the male patients with shigellosis in Taiwan, 2010-2019, and serotype distribution of 109 *S. flexneri* isolates from indigenous cases of shigellosis, 2015-2019.

Of the 153 *Shigella* isolates from male patients with indigenous shigellosis, 33 (21.6%) and 116 (75.8%) were *S. sonnei* and *S. flexneri*, respectively, and 4 (2.6%) unidentified species. An increase of *S. sonnei* isolates was noted during 2015-2016, while *S. flexneri* was responsible for the majority (88.5%, 100/113) of indigenous shigellosis between 2017 and 2019. Serotyping was performed for 109 indigenous *S. flexneri* isolates. The predominant serotype during 2016–2017 was 3a, accounting for 64.7% (22) of 34 *S. flexneri* isolates (Figure 3). However, serotype 2a predominated (67.1%, 47/70) during 2018-2019. All but one case of indigenous shigellosis caused by *S. flexneri* 2a or 3a involved male patients who were either PLWH or MSM.

Antibiotic susceptibility profiles of *S. sonnei* and *S. flexneri* isolates from 149 males with indigenous shigellosis are summarized in Table 3. Before 2015, resistance to ciprofloxacin/levofloxacin or cefotaxime was rarely seen; however, since 2015, the rate of fluoroquinolone susceptibility in *S. sonnei* and *S. flexneri* isolates had declined to 7.4% (2/27) and 40.4% (46/114), respectively. In 2018 and 2019, with *S. flexneri* as the predominant aetiology of indigenous shigellosis, isolates with antimicrobial resistance became prevalent in *S. flexneri*; of note, only one (2.8%) of 36 *S. flexneri* isolates was susceptible to fluoroquinolones.

In terms of the treatment for indigenous shigellosis cases from 2010 to 2014, the overall rate of inappropriate use of empirical therapy was 58.8%; in 2015-2016, 2017, 2018, and 2019, the rates of inappropriate use of empirical therapy remained high. On the contrary, there was an increasing trend in the susceptibility of *S. flexneri* to ampicillin and azithromycin and *S. sonnei* to trimethoprim-sulfamethoxazole (TMP-SMX) (Table 3).

Of the 116 *S. flexneri* isolates identified from male patients with indigenous cases of shigellosis, 7 (6.0%) were resistant to cefotaxime (Table 2), which were also resistant to both ciprofloxacin and ampicillin: 3 identified from MSM and 4 from PLWH. One fatality occurred in a 56-year-old HIV-uninfected male patient with cefotaxime-resistant shigellosis, who presented with fever and diarrhoea that rapidly evolved into septic shock and died of hospital-acquired pneumonia after 27 days of hospitalization.

**Table 2.** Clinical characteristics, outcome, and antimicrobial susceptibility of seven male patients with cefotaxime-resistant *Shigella flexneri* infection.

Diagnosis date	Serotype	Age (years)	Source	MSM	HIV	Antimicrobial susceptibility					Antimicrobial therapy	Outcome
						AZM	CTX	CIP	AMP	SXT		
2014/5/23	N/A	36	Imported	Yes	Yes	N/A	R	R	R	R	AZM	Recovered
2017/10/2	2a	29	Indigenous	Yes	Yes	S	R	R	R	R	CRO	Recovered
2018/8/14	N/A	56	Indigenous	No	No	S	R	R	R	S	LVX	Died
2019/1/17	2a	38	Indigenous	No	No	R	R	R	R	R	CIP	Recovered
2019/5/6	N/A	2	Indigenous	No	No	S	R	I	R	S	CFZ	Recovered
2019/11/21	2a	26	Indigenous	Yes	Yes	S	R	R	R	R	CTX	Recovered
2019/12/18	ND	32	Indigenous	No	Yes	S	R	R	R	R	AZM	Recovered

S = susceptible; I = intermediate; R = resistant; NA = not available; CTX = cefotaxime, CRO = ceftriaxone, CFZ = cefazolin, CI*P* = ciprofloxacin, LVX = levofloxacin, SXT = co-trimoxazole, AZM = azithromycin; N/A = not available, ND = non-typeable.

## Discussion

In this study, a major shift in the epidemiology of the cases of shigellosis was demonstrated by the nationwide surveillance from 1999 to 2019 and a multicentre investigation from 2010 to 2019. During 2010-2014, cases of shigellosis mainly occurred in individuals with foreign travel and paediatric patients, while subsequently, domestically acquired shigellosis has become predominant and was noted mainly in MSM and PLWH since 2015. The latter group of affected patients had longer durations of diarrhoea and hospitalization and had multiple coinfections, which was similar to the findings of a ten-year investigation of shigellosis in England from 2003 to 2013, in which more than half of shigellosis cases aged 16–65 years were male without recent travel and these male patients were mostly MSM [27].

Like shigellosis, HIV infection has been a notifiable disease in Taiwan since 1984. According to the statistics of NDSS, the numbers of indigenous cases of newly diagnosed HIV infection from 2010 to 2019, the period of our retrospective study, are shown in supplementary Table 3. The annual case number of newly diagnosed HIV infection continued to increase until 2018, when a decreasing trend was noted with implementation of programmes of HIV self-testing, pre-exposure prophylaxis, and rapid initiation of antiretroviral therapy within 7 days of confirmed HIV diagnosis. However, we did not find the association between the increasing trends of shigellosis with the trends of newly diagnosed PLWH during the study period.

**Table 3.** Antibiotic susceptibility of *Shigella flexneri* and *Shigella sonnei* isolates from 149 males with indigenous shigellosis.

Drugs	2010–2014	2015–2016	2017	2018	2019
***S. flexneri***					
Ampicillin	0 (0/2)	21.4 (3/14)	22.2 (6/27)	54.1 (20/37)	72.2 (26/36)
Azithromycin	–	33.3 (2/6)	79.2 (19/24)	94.6 (35/37)	97.1 (33/34)
Cefotaxime	100 (2/2)	100 (14/14)	92.6 (25/27)	97.3 (36/37)	88.9 (32/36)
Ciprofloxacin/levofloxacin	100 (2/2)	85.7 (12/14)	74.1 (20/27)	35.1 (13/37)	2.8 (1/36)
Trimethoprim-sulfamethoxazole	50 (1/2)	78.6 (11/14)	88.9 (24/27)	45.9 (17/37)	52.8 (19/36)
***S. sonnei***					
Ampicillin	66.7 (4/6)	100 (18/18)	100 (6/6)	100 (3/3)	–
Azithromycin	–	100 (2/2)	75 (3/4)	100 (3/3)	–
Cefotaxime	83.3 (5/6)	100 (18/18)	100 (6/6)	100 (3/3)	–
Ciprofloxacin/levofloxacin	100 (6/6)	11.1 (2/18)	0 (0/6)	0 (0/3)	–
Trimethoprim-sulfamethoxazole	0 (0/6)	22.2 (4/18)	66.7 (4/6)	100 (3/3)	–

Data are expressed in susceptible percentages (susceptible/total isolates).

Shigellosis is widely known as a highly contagious intestinal infection and often presents as acute dysentery [28]. In our study, 18 cases of shigellosis manifested as chronic diarrhoea, and 83.3% (15/18) of them occurred in either MSM or PLWH. In Taiwan, all individuals with shigellosis can be treated with medical cost reimbursed by the National Health Insurance once diagnosed; therefore, it seems less likely that these cases of shigellosis that manifested as chronic diarrhoea were because of delays in seeking medical attention. We speculate that there might be longer prodromal periods among MSM or PLWH with shigellosis and delays occurred in collecting appropriate clinical specimens for microbiological cultures when they sought care.

In this study, we observed a shift of microbiological characteristics of the *Shigella* species identified from men with indigenous shigellosis, between 2015–2016 when fluoroquinolone-resistant *S. sonnei* predominated, and 2018–2019 when fluoroquinolone-resistant *S. flexneri* 2a predominated. The serotype shift of *S. flexneri* from 3a toward 2a in Taiwan has been demonstrated earlier. Liao *et al.* studied azithromycin-nonsusceptible *S. flexneri* 3a isolates collected during 2015–2016 and demonstrated these isolates belonged to an outbreak sublineage A among MSM with intercontinental dissemination [22,29]. In the United Kingdom, there had been a growing case number of domestic shigellosis due to *S. flexneri* 2a during 2012–2014 [30], and in Iran, the circulation of multidrug-resistant *S. flexneri* 2a was recognized among MSM during 2016–2018 [31]. In conjunction with our findings of a serotype shift of *S. flexneri* from 3a toward 2a in Taiwan since 2018, the international spread of *S. flexneri* is likely ongoing, and further intercontinental collaborations to track and prevent the spread of this prevalent clone are warranted.

As noted earlier, during 2015–2016 there was concurrent emergence of fluoroquinolone-resistant *S. sonnei* and azithromycin non-susceptible *S. flexneri* 3a, both of which were commonly present among MSM and PLWH [21,22]. Previous epidemiologic investigations have recognized several risk factors of shigellosis in recent outbreaks in Taiwan, involving PLWH with high plasma HIV RNA load, poor adherence to antiretroviral therapy and care, oro-anal sex, and chemsex [14,21,22]. Likewise, in our study, MSM and PLWH were prevalent among the indigenous cases of shigellosis since 2015. Less than 50% of PLWH with shigellosis in this study had achieved HIV viral suppression, suggesting poor adherence to antiretroviral therapy or HIV care. Moreover, more than 80% of them had had a history of STDs, and one-fifth of PLWH with shigellosis had ever used recreational drugs. A previous study in England and Wales during 2012–2013 showed that patients infected with *S. flexneri* 3a were mostly MSM; more than half of them were PLWH, had multiple sexual partners by casual encounters and condomless sex, and often had chemsex [12]. These studies and our findings suggest that behaviours that increase the risk of acquiring STDs may also increase the risk of transmission of shigellosis.

While the reasons remain unclear for the evolution of antimicrobial susceptibility of *Shigella* isolates to ampicillin, azithromycin, and TMP-SMX observed in 2015–2019 and more investigations are warranted, the emergence of cefotaxime-resistant *S. flexneri* strains in Taiwan deserves special attention. Prior studies have reported *S. flexneri* isolates with decreasing susceptibility to third-generation cephalosporins in India, China, and Iran [32–34]. Production of beta-lactamases, including *bla*_TEM_, *bla*_OXA_, and *bla*_CTX-M_, was found in these *S. flexneri* isolates. However, in these studies, clinical manifestations and adverse clinical outcomes were not described. In this study, we found that cefotaxime-resistant *Shigella* strains might prove fatal. Surveillance of *Shigella* isolates for emergent antimicrobial resistance and clinical follow-up of patients with shigellosis are needed to inform antimicrobial recommendations for optimal treatment of individuals with shigellosis.

With the increasing antimicrobial resistance, inappropriate use of antibiotics is not uncommon in the treatment of shigellosis in recent years. In our cohort, 48.8% (82/168) patients with indigenous shigellosis received inappropriate antibiotics as the initial therapy, including a fluoroquinolone in 41.5% (34/82) of the cases. Such an emerging threat of ciprofloxacin-resistant *Shigella* isolates may be related to the low resistance barrier of the fluoroquinolone class and the spread of the specific clone among the at-risk populations [35]. Up to now, fluoroquinolones remain the drugs of choice in treating enteric bacterial infections, based on the mainstream guidelines [36–38]. With the ongoing endemic spread of drug-resistant *Shigella* isolates among the at-risk populations, recommendations for empirical antibiotic regimens need to consider the emergence of antibiotic resistance.

There are some limitations in this study. Firstly, the retrospective study of clinical characteristics of shigellosis involved 11 participating tertiary hospitals around Taiwan. We might miss indigenous cases of shigellosis that occurred in mountainous townships or in densely populated institutions in urban areas, who might be treated in local clinics or public health centres. In contrast, after 2015 shigellosis cases involved mostly MSM and PLWH, who were accustomed to seek HIV care at these tertiary hospitals. Furthermore, in PLWH and MSMs other STDs and viral hepatitis cared at tertiary hospitals would be studied concurrently; in contrast, not all paediatric patients had such detailed information. Therefore, selection and information biases were inevitable in our study. Secondly, though we described the changing epidemiology of shigellosis in a ten-year study period, the reason why shigellosis had predominantly affected MSM or PLWH after 2015 remains unclear. At the same time, beginning from 2015-2016, an unprecedented large outbreak of acute hepatitis A that predominantly affected MSM occurred in Taiwan [39,40]. We can reasonably speculate that both shigellosis and hepatitis A were sexually transmissible enteric infections among MSM or PLWH. Thirdly, this study focuses on the epidemiological investigation and clinical comparisons; further molecular tests including multi-locus sequence typing or even whole-genome sequencing are necessary to disclose the relatedness of *Shigella* isolates and to track the route of possible international spread.

Our study has clinical and public health implications. To prevent from shigellosis, people should avoid ingestion of uncooked food and unboiled water. Personal hygiene should be maintained, including hand washing, disinfection of bathroom faucets and door handles, especially before and after approaching diarrheal patients. For MSM, multiple sexual partners, condomless or oro-anal sex should be avoided; wearing latex gloves or dental dam may help protect themselves [41]. The public and private health services should be alerted to the emergence of antimicrobial resistance of *Shigella* spp., for which programmes to promote appropriate use of antibiotics should be reinforced. Novel vaccine strategies, including serotype-based vaccines and conserved antigen vaccines, are regarded as being promising, and there are several vaccine candidates under development or clinical trials [42].

In conclusion, we report the changing epidemiology of shigellosis in Taiwan in 2010-2019. There was a growing number of indigenous cases of *Shigella* dysentery in MSM and PLWH, which often manifested as chronic diarrhoea and led to a longer duration of hospital stay. The predominant strains of shigellosis have shifted from fluoroquinolone-resistant *S. sonnei* to azithromycin non-susceptible *S. flexneri* 3a and fluoroquinolone-resistant *S. flexneri* 2a with the emergence of cefotaxime-resistance *Shigella* species. Physicians should consider such trends of antimicrobial resistance in choosing the optimal treatment for suspected cases of shigellosis.

## Supplementary Material

Supplemental Material

Supplemental Material
