# Supplementary figures and images for: Changing epidemiology of shigellosis in Taiwan, 2010-2019: an emerging threat to HIV-infected patients and men who have sex with men
Source: Emerg Microbes Infect. 2022 Feb 10;11(1):498–506. doi: 10.1080/22221751.2022.2031309 (PMC8855726; doi:10.1080/22221751.2022.2031309)

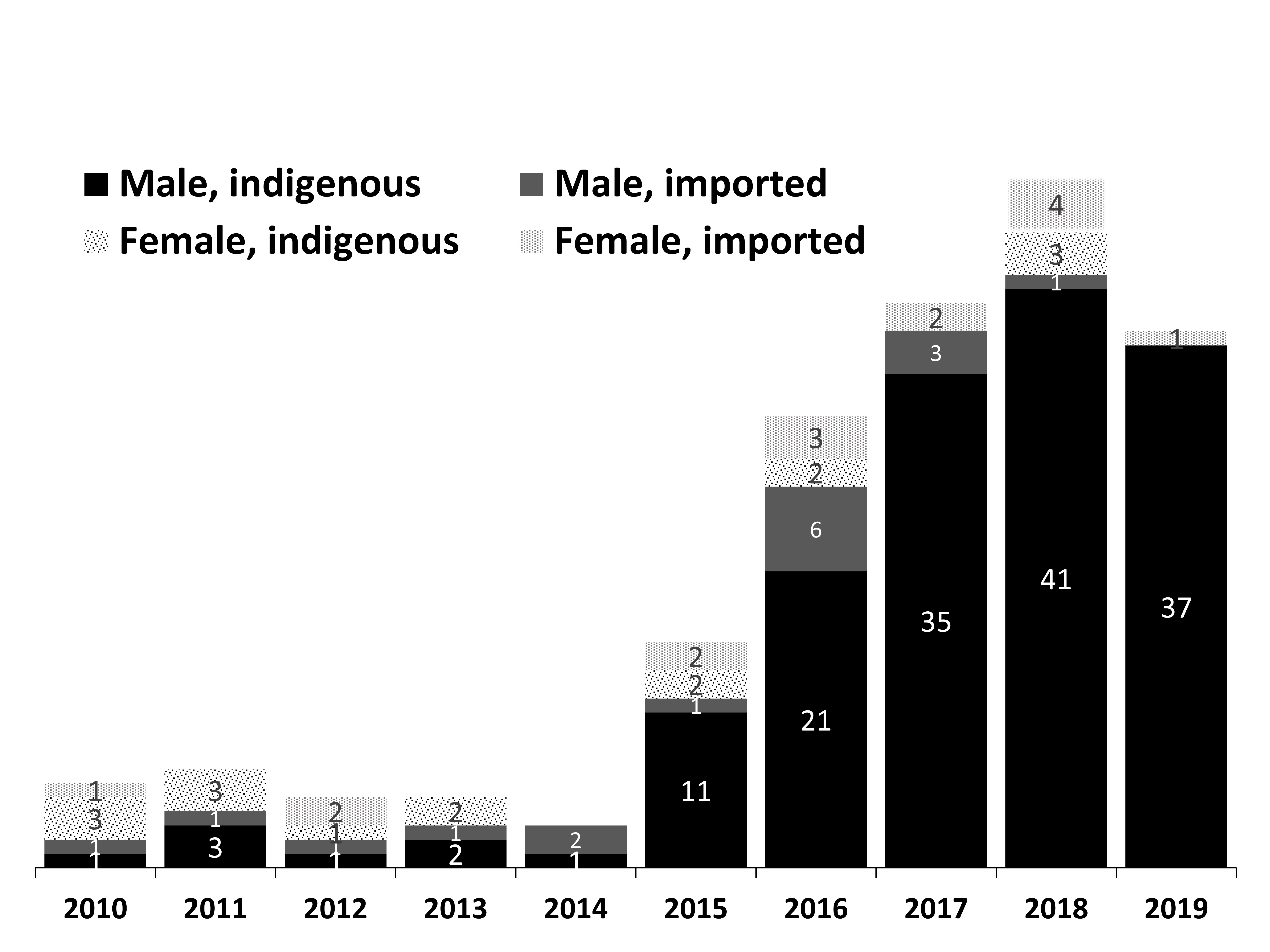

Supplement: Supplemental Material [file TEMI_A_2031309_SM7344.tif]

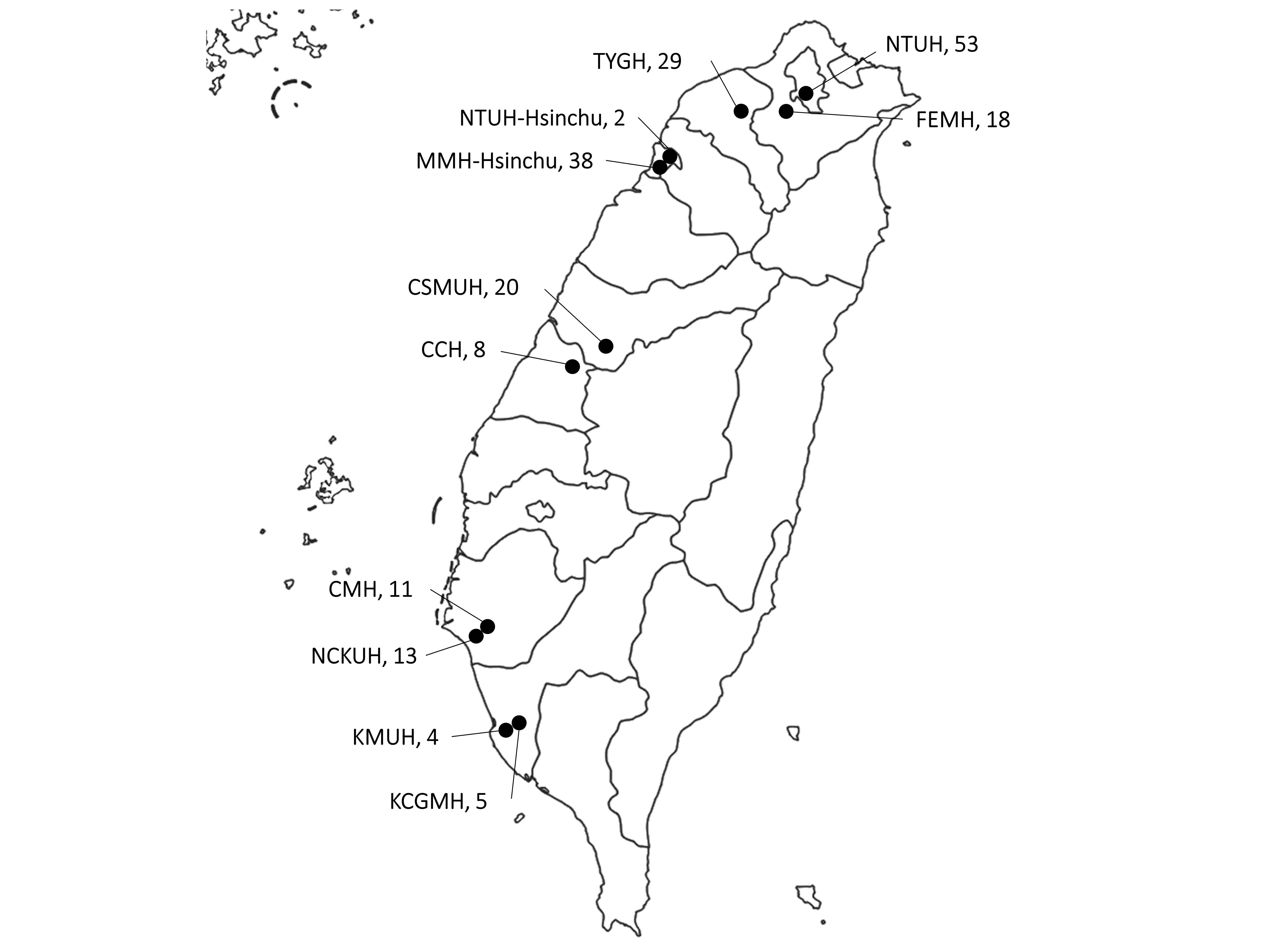

Supplement: Supplemental Material [file TEMI_A_2031309_SM7342.tif]
